# Sketch of the Professional Character of the Late William Lister

**Published:** 1830-05

**Authors:** 


### OBITUARY.

*Sketch of the Professional Character of the late* William Lister, M.D. *formerly Physician to St. Thomas's Hospital.*

This estimable physician, after maintaining a deservedly high reputation in this metropolis for nearly half a century, died at his house in Lincoln's Inn Fields, on the 3d of January, 1830, aged seventy-three years.

Dr. Lister possessed an acute and vigorous understanding, which had early received the culture of a liberal and extended education. His deep and solid attainments, both in philosophy and in the classics, formed an admirable basis for studies more directly of a professional nature. These he afterwards pursued in the university of Edinburgh, with such persevering ardour and success as to acquire a high character for his knowledge of medicine and the collateral sciences. He took an extensive range in study, and always continued to retain an attachment to general science; and it is worthy of remark that, to the very last, he continued to keep pace with the improvements of the day, and, even in chemistry, to make himself intimately acquainted with the rapid progress of discovery. So great a love also did he cherish for classical literature, that, until within a short time of his death, he was accustomed, in the intervals of professional duty, to which he conscientiously devoted a large portion of his time and energy, to recreate himself with the poets and historians of Greece and Rome. Nor did he discover any diminution of interest in the science of mind, on which he continued to read with the same deep attention and eager spirit of inquiry which had characterized the investigations of his early collegiate life.

Notwithstanding, however, this steady attachment to general science and literature, in which his acquirements were not less extensive than profound, Dr. Lister constantly made his profession the principal object of attention. Few individuals, perhaps, have possessed a constitution of mind better adapted for the prosecution of medical inquiry. An acute perception and great power of attention were united with a sound and discriminating judgment, by which he was enabled to view a subject in all its bearings, carefully separating what was essential from that which was merely accidental and adventitious, and generally deducing from the whole a correct and logical conclusion. So thoroughly and patiently, indeed, did this indefatigable physician investigate the more obscure forms of disease, as seldom to have occasion to amend his opinion or retrace his steps. Like his intimate friends, Dr. Baillie and Mr. Cline, he was accustomed to express his view of a case in a few clear, forcible words, and in a manner simple and unadorned, yet calculated to impress the hearer with a conviction of the value and correctness of the opinion.

Dr. Lister's practice exactly corresponded with the clearness and decision of his mind, evincing an equal degree of simplicity and of energy; and thus enabling him to ascertain, with considerable accuracy, the progress of the disease and the effects of the remedies.

Nor would it be proper to omit a special reference to those sterling moral qualities, which were not less conspicuous and influential than his intellectual endowments. Uncompromising integrity and genuine disinterestedness were strikingly observable in his whole character. The welfare of his patients and friends, rather than his own individual interest, appeared to be the predominating principle of action. He had a just conception of what belonged to the character of a physician, and always maintained, by example as well as by precept, the dignity and value of his honourable profession.

With such principles and such conduct, it is not surprising that Dr. Lister should have inspired, in the minds of those who had the privilege of his friendship, a high degree of respect and attachment; although, from a rooted aversion to every thing like pretension and display, his manner may have appeared to strangers cool and unattractive. Those, however, who knew him intimately had abundant proofs of the tenderness and depth of his feelings.

With a mind so well stored and disciplined, and with opportunities and habits of observation so favorable to research, it is to be regretted that Dr. Lister should have written comparatively little. The specimens of biography given in the Gentleman's Magazine for November 1817, and October 1823, containing short memorials of two of his most beloved and intimate associates, viz. Dr. Wills and Dr. Baillie, sufficiently prove how admirably he was qualified for literary undertakings.

But to the most able and diligent, as well as to others, "there is a time to die." Dr. Lister contemplated that important change with remarkable composure. During the last thirty years of his life, indeed, he had suffered repeated attacks of angina pectoris, and had a constant persuasion of being himself the subject of organic disease about the heart. Of this settled and deliberate conviction he could not divest his mind, notwithstanding the remonstrances of his brethren, especially of his intimate friend Dr. Wells, who laboured to persuade him he was merely hypochondriacal: yet the post-mortem appearances decisively prove that Dr. Lister's usual judgment did not forsake him even in the consideration of his own individual case.

Among the papers examined after his death, a memorandum was found, dated December 20, 1821, in which he details the principal symptoms of his complaint, and his opinion of their nature, concluding with the following direction: "To ascertain the truth of the above conjecture, and to recommend the practice of post-mortem examinations by an example in my own person, I desire that my excellent friend, Mr. J. H. Green, may be requested to make a complete examination of me as soon after my death as he thinks desirable, and to furnish my son Nathaniel* with a statement of all he observes." In accordance with this request, an accurate inspection was made by Mr. Green, which remarkably confirmed the opinion which the deceased had entertained of the nature of his disease. The valves of the aorta, as well as various portions of the aorta itself, were ossified, as were also the coronary arteries. The mitral valves were also partially ossified, and the tricuspid passing into the same state. There was hypertrophy of the left ventricle; and adhesions had formed between the heart and pericardium. A large quantity of serum was contained in the cavities of the pleura. The internal carotid arteries were ossified, and the vertebral arteries thickened.

Notwithstanding occasional paroxysms of agonizing pain, Dr. Lister steadily pursued his usual avocations, and actually visited his patients until two days preceding his death. He had suffered, however, exceedingly during the severe weather of January last, both from difficulty of breathing and general uneasiness about his chest. Towards the evening of Tuesday, symptoms of effusion more distinctly appeared; and on the morning of Wednesday, surrounded by his numerous and affectionate family, and in the full possession of his mind, this venerable man gradually ceased to breathe.

Febr

T. H. B.

*Brunswick square; April 7th,* 1830.

---

The celebrated S. T. von Soemmering, who maintained so high a rank as an anatomist and physiologist, departed this life at Frankfort, on the 2d of March, in the seventy-sixth year of his age.

---

### BIBLIOGRAPHICAL NOTICES.

* Then a student in medicine, but now M.D.

