# Erratum

**Published:** 1830-06

**Authors:** 


ERRATUM in the last Number. In the Memoirs of the late Dr. Lister, page 471, line 17, for "January" read "February."

